# Heated Tobacco Products: A Review of Current Knowledge and Initial Assessments

**DOI:** 10.3389/fpubh.2019.00287

**Published:** 2019-10-10

**Authors:** Nadja Mallock, Elke Pieper, Christoph Hutzler, Frank Henkler-Stephani, Andreas Luch

**Affiliations:** Department of Chemical and Product Safety, German Federal Institute for Risk Assessment (BfR), Berlin, Germany

**Keywords:** heated tobacco products, emissions, harm reduction, risk assessment, tobacco smoke

## Abstract

The health risks of tobacco smoking have been documented in numerous studies and smoking rates have declined in developed countries over the last 50 years. Today, we know that cigarette smoking is the major cause of preventable deaths due to tobacco smoke induced diseases. As a consequence of an increased awareness of smoking-related health risks, heated tobacco products (HTPs) are marketed as reduced toxicant alternatives to conventional tobacco products. Manufacturers claim that levels of toxicants and hazardous compounds are significantly reduced, implying that inhalation of the modified aerosol is less harmful compared to conventional cigarettes. In this manuscript, previous assessments of HTPs are briefly summarized, including a short discussion on challenges with the adaption of standard analytical methods used for tobacco smoke. The reliability of analytical data is important for risk assessment approaches that are based on reduced toxicant exposure. In order to assess a putative reduction of health risks, an integrated study design is required that should include clinical studies and epidemiology data. One manufacturer applied for a classification as a Modified Risk Tobacco Product (MRTP) in the United States, based on extensive toxicological studies that have also been published. However, data are not yet sufficient for a reliable assessment or recognition of putatively reduced health risks. Challenges regarding a classification in Europe are also discussed briefly in this review.

## Introduction

Although most smokers are aware that tobacco smoking is harmful to their health, it is still the leading cause of premature death worldwide and claims the lives of more than 6 million people every year due to cancer, heart disease, stroke, chronic bronchitis, and emphysema (1–4). A recent study has shown that tobacco smoking increases not only the risk for lung cancer, but also for at least 17 different malignant diseases in humans (5); therefore, successful tobacco control can save millions of lives. With the Framework Convention on Tobacco Control (FCTC), the World Health Organization (WHO) has initiated a comprehensive tobacco control strategy (6). Articles 9 and 10 of the FCTC include specific policy measures to curb tobacco use by regulating the ingredients and the emissions of tobacco products. The overall aim is to decrease toxicity, addictiveness, and appeal to consumers. Parties of this convention have committed themselves to restrict the supply and demand of tobacco products through a wide range of policies and measures. Although FCTC was successfully applied to conventional tobacco products, uncertainties remain on how to cover novel products. In October 2018, Conference of Parties (COP) 8 explicitly proposed to extend the scope of the according legislations to Heated Tobacco Products (HTPs) (7).

**Figure 1:**
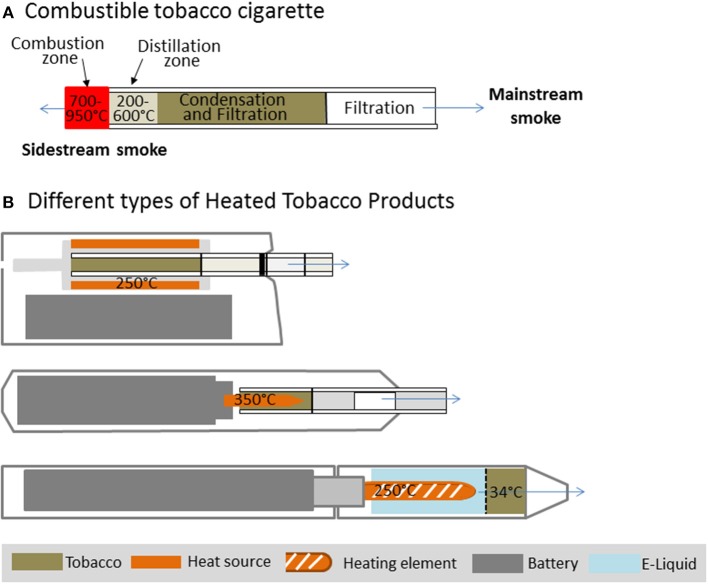
Temperature zones in a combustible cigarette **(A)** in comparison to different Heated Tobacco Products **(B)**.

The chemical complexity of cigarette smoke depends on heating conditions inside the lit cigarette. In a conventional cigarette the burning of tobacco leads to combustion at temperatures up to 700–950°C during puffs (see Figure 1A). While combustion is limited to the tip of a burning cigarette, pyrolysis and thermal decomposition occur in the oxygen deficient distillation zone. In this part of the cigarette temperatures decrease from 600 to about 200°C. The majority of smoke toxicants are generated here. Below 350°C, condensation of less volatile compounds generates a dense aerosol consisting of growing droplets and solid particles (8). As a consequence, cigarette smoke consists of “particulate” and “vapor” phases. The mainstream smoke comprises all constituents inhaled during a puff. One way to reduce the exposure to harmful and potential harmful compounds (HPHCs) in the mainstream smoke of tobacco products is to lower the temperature applied to the tobacco. This approach had previously been tried but could not find acceptance on the market as the technology was not yet advanced (9, 10).

Recently, a new generation of HTPs has been introduced to the market which differs widely in product design and temperatures applied to the tobacco. In some devices the tobacco is heated up to 350°C via an electrical heating source (11, 12) or different sources like carbon (13), whereas in other devices vapor is passed through the tobacco and extracts compounds including flavors and nicotine at lower temperatures (14, 15). Three different device designs which are currently present on the market are displayed in Figure 1B. These products contain real tobacco that does not undergo a self-sustaining exothermic combustion.

## Emissions

In accordance with the principle of temperature dependence of HPHC generation in tobacco products, the question of reduced HPHC levels in the emissions was raised. While manufacturers provided the initial studies (15–18), more and more independent investigations have now been published for commercially available products (19–29). These studies were focused on levels of well-known HPHCs in comparison with other tobacco products. Analyzed HPHCs were adopted from the FDA preliminary HPHC list (30) and recommendations by the WHO Study Group on tobacco product regulation (TobReg) (31). Important carcinogens, such as aldehydes and volatile organic compounds, were found to be reduced by about 80 to over 99% (25). The lowest reduction with only about 80–90% was reported for acetaldehyde, classified as possibly carcinogenic to humans by the International Agency for Research on Cancer (IARC) (32). Toxicants like tobacco-specific N-nitrosamines (TSNAs), formed primarily during curing and processing of tobacco rather than by combustion, were also present in the filler of HTP consumables. However, compared to cigarette mainstream smoke TSNA levels were reduced by about 80–90% (20). Metals like cadmium and mercury are taken up by the tobacco plants and are therefore naturally present in products that contain tobacco (33, 34). Again, levels were reduced in HTP devices. Whereas cadmium was below detection limit, indicating a reduction of over 99% (16, 17), reduction of mercury was ~75% as published for one device (17). Polycyclic aromatic hydrocarbons (PAHs) and carbon monoxide are typical products of incomplete combustion. Although reduced by more than 90%, they are still present in HTP emissions (17). Other substances, such as propylene glycol, glycidol, acetol, and 2-propen-1-ol have been shown to be elevated in comparison to the combustible reference cigarette in at least one device, due to the higher amount of humectants in the tobacco filler of the HTP consumable (35). Influence on indoor air quality was assessed by the manufacturers and found to be significantly reduced compared to combustible cigarette smoke (14, 36, 37). Concerns for the use in small and poorly ventilated rooms have been raised by an independent group (38).

Reliability and reproducibility of emission data is a crucial factor for a subsequent risk assessment. To benefit most from the increasing pool of independent studies, a common standard for measurements should be agreed on. The first open question arises regarding the machine puffing protocol. There are different arguments for and against various standard protocols, such as ISO (39) or Health Canada Intense (40). Since some of these devices turn off by themselves after a certain time, a smoking regimen with a higher frequency like HCI can help to collect enough material per consumable to pass thresholds set by the analytical instruments. However, the HCI regime could lead to overestimated reductions, due to blocked filter ventilation in conventional or reference cigarettes. Since this modification results in higher toxicant levels in cigarette smoke, the calculated relative reductions of toxicants in the emissions appear bigger. A new puffing protocol, especially tailored for HTPs, would be possible as well. Importantly, these standard protocols do not mimic average smoking behavior and are not meant to provide a realistic estimate of exposure (41). The purpose of defined smoking regimes is to provide standards to compare key parameters of different products when analyzed in different laboratories. However, recent investigations of the puffing topography (42, 43) might suggest further refinements for a better adoption of machine smoking to HTP. ISO/TC126 and CORESTA have started to work on standardized methods.

Since aerosols of HTPs contain a comparatively high proportion of water, standard analytical procedures cannot be easily applied here. Water is trapped on the glass fiber filter and therefore accounts for the total particulate matter (TPM). When the filter is processed further, water loss can occur leading to a reduced analyzed water content. Although not a toxicant, water becomes important when the nicotine-free dried particulate matter, commonly referred to as “tar,” is calculated by the subtraction of water and nicotine from TPM (44), though the tobacco industry has developed methods in order to avoid water loss (45, 46). When special equipment is required, implementation as a standard method by independent laboratories becomes difficult. Despite these technical challenges, industry and independent laboratories have come to mostly comparable results when using standard procedures that were designed for the analysis of conventional cigarettes. This indicates that these procedures could be a basis for dedicated analytical standards for HTPs.

## Risk Assessment Approaches

As discussed, most harmful substances that are known to occur in cigarette mainstream smoke were shown to be lowered by one or two orders of magnitude in HTP emissions. Promoted by the manufacturers, there are discussions if this means a reduction of health risks for HTP consumers followed by controversies whether HTPs can be seen as part of harm reduction strategies. The underlying idea of harm reduction strategies in tobacco control is that the damage caused by tobacco consumption should be at least reduced when it cannot be prevented. Toxicant reduction is not necessarily linked to decreased health risk. Although levels of tar had decreased in combustible cigarettes since the 1950 by nearly two thirds, this was not correlated with corresponding decrease in lung cancer incidences (47). One strategy to assess modified health risks is to compare the tumor potencies of aerosols, as previously applied by Fowles and Dybing to rank the relevant carcinogens and toxicants in cigarette smoke. These calculations are based on individual detection levels in mainstream smoke and on cancer potency factors as indicators of the carcinogenic risk for each smoke constituent (48). The German Federal Institute for Risk Assessment confirmed in its previous study substantially reduced toxicant levels for selected HTPs and provided an initial assessment in 2017 (49). The profound reduction (>99%) of key carcinogens according to Fowles and Dybing, such as benzene and 1,3-butandien, as well as substantial overall reduction of toxicants is expected to affect health risks, if people abstain completely from other tobacco products. Nicotine levels are still in the range of conventional cigarettes, limiting the risk to switch back to conventional smoking tobacco (25). In a detailed modeling assessment, Stephens compared relative harmfulness of different nicotine products with a model based on exposure data and cancer potencies. The calculated lifetime cancer risk of the HTP, using one data set by the manufacturer, was one to two orders of magnitude lower compared to combustible cigarettes but higher compared to e-cigarettes (50). Lachenmeier et al. calculated the combined margin of exposure (MOE) for the HTP and for combustible cigarettes (51). The obtained ratio between exposure and toxicity effect levels, which could be interpreted as a “safety buffer” (52), was 10-fold higher for the HTP as compared to combustible cigarettes (51). As noted by Stephens, these models only consider toxicants levels and neglect particle effects (50). In addition, there is growing consensus that a complete switch to HTP can reduce toxicant exposure, as confirmed in recent investigations on biomarkers of exposure in smokers (53–57). Haziza et al. reported reductions of 51 to 96% for selected HPHC-related biomarkers over a 90-days ambulatory study. However, compliance of participants was decreasing over the ambulatory period, suggesting that relapse to tobacco and/or dual use could counteract potential benefits in real life settings (54). During two 90-days studies, biomarkers of potential harm were additionally assessed (58, 59). The results of longer switching studies to detect significant reductions of biomarkers of potential harm are anticipated (60).

**Figure 2:**
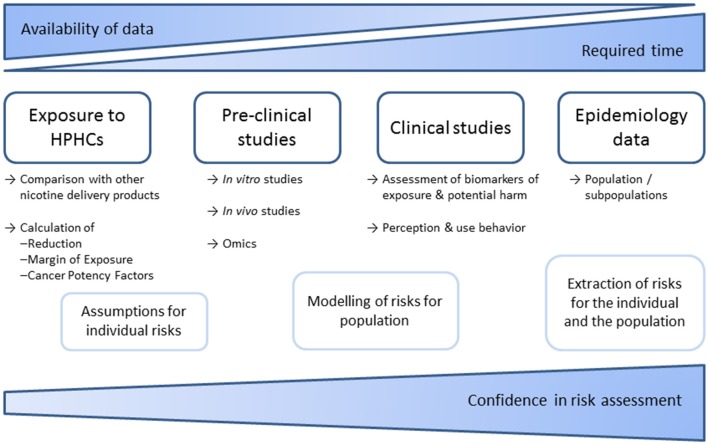
Schematic overview of required data and studies to facilitate risk assessments of tobacco products. Epidemiological data are most conclusive but can usually only be used retrospectively. Therefore, risk assessments rely on models that consider emissions, pre-clinical and clinical studies. Meanwhile numerous studies on smoke chemistry and *in vitro* toxicology have been published by industry and independent researchers. In contrast, *in vivo* and clinical studies are far more complex. No sufficient independent data are available.

In the United states, the Family Smoking Prevention and Tobacco Control Act (61) requires tobacco products to not only “*significantly reduce harm and the risk of tobacco-related disease to individual tobacco users*” but also to “*benefit the health of the population as a whole taking into account both users of tobacco products and persons who do not currently use tobacco products*” in order to market that product with modified risk claims in the United States. The required scientific evidence for defined claims and additional data that have to be provided by the applicant are described by the FDA in detail in a guidance document (62). Scientific standards for analysis of potential Modified Risks Tobacco Products were also outlined by the Institute of Medicine in 2012 (63). Required data (summarized in Figure 2) include a comprehensive analysis of smoke chemistry (64) as well as data on specified biomarkers of exposure. There is a framework for preclinical studies, proposing *in vitro* tests of genotoxicity, oxidative stress, and inflammation. The *in vitro* test battery comprising assays for bacterial mutagenicity, mammalian cytogenetics/mutation, and mammalian cytotoxicity, that has been suggested by a CORESTA task force in 2004 (65), has been conducted by the manufacturers (17, 18, 66–70). Some *in vitro* tests can specifically address smoking related adverse effects, as biphasic culture of airway epithelial cells or assays on endothelial activation as conducted by the manufacturers (13, 71–74) and independent researchers (75, 76). Further, 3D *in vitro* cultured lungs tissues are now available by several commercial suppliers. Consequently, the necessity for animal testing of tobacco products should be questioned, in line with a general shift of focus in modern toxicology (77). In some countries including Germany, animal studies have been prohibited for tobacco products. However, animal studies have been conducted by the tobacco industry (78–81) and independent researchers (82). To address public health questions, population models have been applied (83–86) and publically discussed (87).

In Europe, toxicological assessments of tobacco products are aimed to exclude elevated risks in relation to conventional products, but not to confirm less hazardous product properties. As long as relevant adverse effects cannot be excluded, even modified health risks still remain an issue of concern. In contrast to the United States, products can be placed on the market more easily. Consumers who use these products need to accept all characterized and not yet identified health risks. Also manufacturers might attempt to gain classification as “smokeless tobacco,” resulting in less stringent health warnings. In public perception, this could probably be understood as an official acknowledgment of reduced health risks. Such acknowledgment would be premature from the perspective of risk assessment. In the USA, the assessment framework is required to acknowledge reduced/modified risks, if manufacturers can support their claims. Consequently, additional issues, as for example risk perception and communication, behavioral assessments of addictiveness or clinical studies (63) need to be considered.

In May 2017, one manufacturer submitted a Modified Risk Tobacco Product Application (MRTPA) for his HTP (88) and in January 2018, the Tobacco Product Scientific Advisory Committee (TPSAC) met to give a recommendation. Due to the lack of human studies, TPSAC was not convinced to support the statement “*Scientific studies have shown that switching completely from cigarettes to the IQOS system can reduce the risks of tobacco-reduced diseases*,” although potential is seen. The relevance of the animal studies to human smokers has been questioned (89). Two 90-days studies as mentioned above (58, 59) did not demonstrate a relevant reduction in biomarkers of potential harm in regard to inflammation and lung function (90). This could also be linked to the continual inhalation of nicotine and remaining toxicants. Reductions of biomarkers of potential harm were also low in the smoking abstinence groups, possibly due to the short study period. Biological relevance needs to be demonstrated with longer exposure studies. However, biomarkers of exposure that have been assessed in various studies were shown to be reduced similarly to cessation level (35), especially markers that are relevant for carcinogenic risks. The less strong claim “*Switching completely to IQOS presents less risks of harm than continuing to smoke cigarettes*” has therefore been supported by about half of the committee members (89). While the evidence has mostly been seen as strong enough to support a reduced exposure claim, the link to morbidity and mortality has not been seen to be adequately demonstrated (89). The final decision on the MRTPA has not been made by the FDA yet, however the first HTP was authorized in April 2019 for sale, without modified risk status. In Europe, it is widely accepted that current HTPs do not bear additional or other health risks in relation to conventional products. European legislation does not define a modified risk classification. On the contrary, information on the product and package, as well as presentation must not imply reduced hazards compared to any other tobacco product. Although a risk-benefit assessment is required for new tobacco products, permission on the market does not depend on modified risks.

Although a 99% reduction of some major carcinogens is expected to affect health risks, the magnitude or relevance of such putative reduction is not yet clear. A benefit is likely seen for especially the subset of long-term smokers that are unable to quit or to switch to another nicotine source with less HPHC exposure. However, referring back to the tumor potency models, it should be kept in mind that substantial and relevant health risks are still present. Consequently, HTPs should not be the first option to decrease smoking-associated harm.

## Author Contributions

NM prepared the draft manuscript. EP and FH-S contributed sections to the draft. NM, EP, CH, FH-S, and AL created the concept for this article and contributed to manuscript revision and approval of the final version.

### Conflict of Interest

The authors declare that the research was conducted in the absence of any commercial or financial relationships that could be construed as a potential conflict of interest.
